# Isotope Dilution Analysis for Particle Mass Determination Using Single-Particle Inductively Coupled Plasma Time-of-Flight Mass Spectrometry: Application to Size Determination of Silver Nanoparticles

**DOI:** 10.3390/nano13172392

**Published:** 2023-08-22

**Authors:** Maite Aramendía, Diego Leite, Javier Resano, Martín Resano, Kharmen Billimoria, Heidi Goenaga-Infante

**Affiliations:** 1 Department of Analytical Chemistry, Aragón Institute of Engineering Research (I3A), University of Zaragoza, Pedro Cerbuna 12, 50009 Zaragoza, Spain; dih.egodh@gmail.com (D.L.); mresano@unizar.es (M.R.); 2 Department of Computer Sciences and Systems Engineering (DIIS), Aragón Institute of Engineering Research (I3A), University of Zaragoza, C/Mariano Esquillor SN, 50018 Zaragoza, Spain; jresano@unizar.es; 3 National Measurement Laboratory, LGC, Queens Road, London TW11 0LY, UK; kharmen.billimoria@lgcgroup.com

**Keywords:** single-particle ICP-MS, time-of-flight mass spectrometry, isotope dilution analysis

## Abstract

This paper describes methodology based on the application of isotope dilution (ID) in single-particle inductively coupled plasma time-of-flight mass spectrometry (spICP-ToFMS) mode for the mass determination (and sizing) of silver nanoparticles (AgNPs). For this purpose, and considering that the analytical signal in spICP-MS shows a transient nature, an isotope dilution equation used for online work was adapted and used for the mass determination of individual NPs. The method proposed measures NP isotope ratios in a particle-to-particle approach, which allows for the characterization of NP mass (and size) distributions and not only the mean size of the distribution. For the best results to be obtained, our method development (undertaken through the analysis of the reference material NIST RM 8017) included the optimization of the working conditions for the best precision and accuracy in isotope ratios of individual NPs, which had been only reported to date with multicollector instruments. It is shown that the precision of the measurement of these ratios is limited by the magnitude of the signals obtained for each NP in the mass analyzer (counting statistics). However, the uncertainty obtained for the sizing of NPs in this approach can be improved by careful method optimization, where the most important parameters are shown to be the selection of the spike isotopic composition and concentration. Although only AgNPs were targeted in this study, the method presented, with the corresponding adaptations, could be applied to NPs of any other composition that include an element with different naturally available isotopes.

## 1. Introduction

Single-particle inductively coupled plasma mass spectrometry (spICP-MS) emerged in the past decade as a powerful tool for nanoparticle (NP) characterization in suspensions and has been gaining popularity in the last years for several reasons [1,2]. SpICP-MS is highly sensitive, does not require extensive sample preparation and has the capability of characterizing NPs in a fast way. It provides information on NP elemental composition, particle size and NP size distribution (as long as some knowledge of the NP shape and density is available or assumed) as well as particle mass and number concentration at low, environmentally relevant levels (10^6^–10^8^ part·L^−1^) [3]. Additionally, it can provide information on the concentration of ionic species coexisting with the NPs in the same dispersion. In the past few years, this working methodology has passed from a developmental to an application stage, and many papers have been published dealing with NP determination in food, cosmetics, biological tissues, animal models, soils or waters, among others [4]. However, there are still some aspects that need further attention, especially concerning the validation of both particle size and particle number concentration determination in real, complex matrices.

When determining NP mass and, then, size in complex samples, spICP-MS can suffer from matrix effects, which degrade the quality of measurements. As for conventional ICP-MS analysis, these effects can be categorized into spectral overlaps or nonspectral interferences, and each one shows particular consequences for this working methodology.

On the one hand, the occurrence of matrix-induced spectral interferences in spICP-MS results in elevated background signals, directly impacting the limit of detection in the NP size (LOD_size_) determination. In these cases, the use of ICP-MS instruments with collision/reaction cells has proven useful for improving these LOD values [5,6].

However, the presence of the matrix can also induce nonspectral interferences that compromise calibration. The most common calibration strategy deployed for spICP-MS consists of using an appropriate method to determine sample transport efficiency to the ICP (TE; the relationship between the detected and aspirated element mass) [7] and generating a mass–flux calibration curve with a set of standard solutions of the analyte in ionic form. With this method, the matrix present in the sample suspensions can affect ICP-MS detection sensitivity and/or sample transport efficiency, causing bias for both the sizing and counting of the NPs unless specific measures are taken for dealing with this problem [8,9,10,11].

In this regard, calibration with NP standards of known size [12] or the use of sample introduction systems with 100% TE [13,14] are alternative possibilities, potentially less affected by matrix effects [15]. However, the use of these strategies is limited by the availability of the NP standards or the alternative introduction devices [16], and anyway, not all of them truly reach 100% TE [17]. Matrix effects can also be alleviated by sufficient sample dilution, although when the NP concentration in complex sample dispersions is already very low, this is not always practical. There are other, more general, calibration strategies dealing with these problems that have been recently adapted for use with spICP-MS, including matrix-matching [9] (as long as the matrix composition is known), standard addition [11] (usually requiring longer analysis times per sample) and internal standardization [18]; these are better or less suited depending on the application.

In any case, and among all strategies available for dealing with matrix effects in ICP-MS [19], isotope dilution analysis (IDA) is generally regarded as the most robust and effective, providing higher accuracy and lower uncertainty over other calibration methods. IDA relies on the intentional alteration of the isotope abundances of an endogenous element in a given sample by the addition of a known amount of an enriched isotope of the same element (spike) [20]. Calculations are based on the measurement of the modified isotope ratios in the mixture “sample plus spike”, which implies that, for the proper application of IDA, it is necessary that two or more isotopes of the analyte element can be measured free of spectral interferences.

This prerequisite is not easy to fulfil for spICP-MS, as with the most commonly used sequential ICP-MS instruments (equipped with either quadrupole or single-collector sector field spectrometers), because only one isotope can be monitored on the time scale of the signal generated by one NP (300–500 µs) [21,22]. As a consequence, only two papers in the literature have reported the application of IDA to spICP-MS with sequential instruments after making some approximations for overcoming this limitation. In the work of Telgmann et al. [23], the average 107/109 isotope ratio for a distribution of silver NPs was calculated for the most frequent NP size by ratioing the mean ^107^Ag^+^ and ^109^Ag^+^ signals of the NP frequency histogram obtained by Gaussian fitting. These ratios were introduced in the IDA equations to obtain the most frequent size of the NP distribution. This working strategy allows for rapid size determination of suspensions of NPs with a distinct size in complex matrices, but information on the particle size distribution is not available.

This problem was circumvented by Sötebier et al. [24] by using a modification of the postcolumn species-unspecific IDA equation [25,26]. In the case of spICP-MS, the transient nature of the signal is given by the detection of the individual NPs over the constant signal of the spike. To measure the isotope ratios for every NP event needed for calculations, the authors used a ^109^Ag-enriched spike (close to 100% ^109^Ag) at a sufficiently high concentration as to be able to consider that the contribution of the NP intensity to the signal at *m/z* = 109 was negligible, so that the intensity at this *m/z* was relatively constant from one measurement cycle to the next. On the other hand, NPs were detected at *m/z* = 107 since the contribution of the spike was really low at this *m/z* value. With this approximation, it was possible to determine 109/107 ratios for every NP event, thus achieving information on the particle size distribution for the sample analyzed. As a drawback, the long measurement cycles that had to be used for monitoring both silver isotopes (22 ms) resulted in only 45% of the NP events being effectively detected at *m/z* = 107, so that long acquisition times (up to 10 min per sample) were necessary to compensate for this fact.

From the foregoing account, the availability of a simultaneous ICP-MS system with a time resolution in the micro- or millisecond range would offer a promising approach towards overcoming the problems mentioned above. An instrument of this sort, based on the use of a time-of-flight (ToF) spectrometer, was introduced as a prototype in 2013 [27] and was made commercially available in 2017 [28]. Due to the (quasi) simultaneous detection of the entire *m/z* spectrum with this instrument, multielemental (or multi-isotopic) information in single nanoparticles becomes available, thus enabling the use of IDA for calibration in single-particle events. However, only one paper to date has reported the use of IDA with spICP-ToFMS [29]. This work was based on the combined use of a microdroplet generator, for avoiding the need to determine the transport efficiency, and isotope ratio determination, needed for calculations performed on average values from histogram Gaussian fits and not on a particle-to-particle basis, due to the large variability detected for the ratios in the latter working mode. Indeed, deploying this calibration methodology on a particle-to-particle basis implies accurate and precise determination of isotope ratios for individual nanoparticles in the aqueous suspensions, which, to the best of our knowledge, has only been reported with multicollector-ICP-MS instruments [30,31].

This work evaluates the performance of ICP-ToFMS in spICP-MS mode combined with IDA to determine the mass and derived size of individual nanoparticles. Method development, including accurate and precise determination of isotope ratios for individual nanoparticles, was undertaken through analysis of the reference material NIST RM 8017 (Ag NPs). The particle mass obtained with this methodology was converted to particle size by considering spherical particle geometry, the Ag density and mass fraction. The method accuracy was evaluated by comparison with the reference values reported for mean Ag particle size in the NIST RM 8017 certificate.

## 2. Materials and Methods

### 2.1. Reagents, Solutions and Materials

Ultrapure water (resistivity 18.2 MΩ, 25 °C) was obtained from an Elga water purification unit (Elga, Marlow, Buckinghamshire, UK). For ionic calibration of the conventional spICP-MS approach with external calibration, appropriate dilutions were daily prepared from a silver stock solution of 984.54 mg·L^−1^ (Romil, Cambridge, UK). For AgNP characterization by IDA, a ^109^Ag-enriched standard solution with a certified Ag ionic concentration of 9.97 µg·g^−1^ in 2% HNO_3_ media and an isotopic abundance of 99.8% for ^109^Ag and 0.2% for ^107^Ag was purchased from ISC Science (Oviedo, Spain). Trisodium citrate tribasic dihydrate with purity higher than 99.5% w·w^−1^ was purchased from Sigma Aldrich (Gillingham, UK). All the ionic solutions and AgNP dispersions (with or without the ^109^Ag spike) were prepared in 1 mM trisodium citrate media.

For transport efficiency (TE) determination and NP characterization, AgNP reference material RM 8017 was purchased from NIST (Gaithersburg, MD, USA). This material contains polyvinylpyrrolidone-coated silver nanoparticles with a nominal diameter of 75 nm. The certified diameter value (measured by TEM) was 74.6 ± 3.8 nm, and the reference silver mass value was 2.162 ± 0.020 mg. This material was reconstituted with 2 mL of ultrapure water, resulting in a silver mass concentration of 1.072 ± 0.010 mg·mL^−1^. The dynamic mass flow approach reported elsewhere [32] was used to determine the particle number concentration of the AgNP dispersions used in this work, resulting in a number concentration of 4.66 × 10^14^ particles L^−1^.

### 2.2. Instrumentation

The characterization of inorganic nanoparticles using spICP-MS was carried out with a Tofwerk icpTOF 2R instrument (Tofwerk, Thun, Switzerland). This instrument is a combination of the ion/optics, collision/reaction cell, gas and vacuum systems of an iCAP Qc from Thermo Scientific (Thermo Fisher Scientific, Bothell, WA, USA) and a notch filter and TOF mass analyzer from Tofwerk. This instrument is designed with an orthogonal acceleration configuration [28]. The instrument is equipped with two software packages: TofPilot (version 2.11.5), for the setup and configuration of the ICP-TOF system, and Tofware (version 3.2), for data processing.

With this instrument, the mass range spectra from ^14^N^+^ to ^254^UO^+^ can be obtained simultaneously under two different acquisition data modes, namely, continuous and triggering modes, whose main characteristics are covered in Section 3.2.1. In this work, all experiments were conducted in continuous mode, for which the fastest rate of detection is 1000 Hz (33 ToF extractions per mass spectrum) and hence, the minimum integration time for the icpTOF 2R is 2.7 ms. According to this limitation, 3 ms integration time was used throughout this study.

The sample introduction system comprises a Micromist nebulizer (400 μL min^–1^) and a Peltier-cooled Scott-type spray chamber. For AgNP characterization, the collision/reaction cell was not used, as the spectral interferences for ^107^Ag^+^ and ^109^Ag^+^ are expected to be negligible. The sensitivity and the mass calibration were adjusted daily in order to achieve the optimal operating conditions of the equipment. The working conditions finally selected are summarized in Table 1.

### 2.3. Methodology for the Characterization of AgNPs by spICP-ToFMS and IDA

For the mass determination of a single AgNP detected by the IDA approach, Equation (3), described in detail in Section 3.1, was used. As will be treated in detail in Section 3, application of this equation requires the accurate and precise measurement of different parameters for each sample, and the protocol described below was followed for achieving this goal.

#### 2.3.1. Determination of Sample Flow (f)

Before analysis, the sample uptake flow f was measured in order to minimize the introduction of systematic errors in the TE calculation and in the AgNP characterization by IDA, as this parameter is critical for both calculations. For this purpose, a vial containing a 2.5 µg L^−1^ ionic Ag solution in 1 mM citrate media was placed on an analytical balance placed next to the ICP-ToFMS. After system stabilization, the weight of the vial was registered every 3 min for 15 min. Each registered weight was plotted against monitorization time, resulting in a linear sample uptake mass flow graph. The slope of the linear regression obtained is the sample flow (in g·min^−1^). This procedure was repeated every day before analysis was carried out.

#### 2.3.2. Recalibration of Mass Spectra

In addition to the general mass calibration performed daily at the beginning of the working session, recalibration of the mass spectra was performed at the beginning and at the end of each analysis batch, using 2.5 µg L^−1^ Ag ionic standards with natural isotope composition in 1 mM citrate matrix. This recalibration was performed to correct for potential mass-calibration drifts of Ag isotopes [28]. In each batch, the measurements of the TE, calibration standards for all operations described in this protocol and IDA samples were carried out. Recalibration of mass spectra was carried out in data acquisition with the Tofware data postprocessing software (version 3.2).

#### 2.3.3. Determination of the Mass Discrimination Factor (K Factor)

In order to correct for mass bias between the theoretical and the experimental (measured) isotope ratio, it is necessary to determine the mass discrimination factor (which we called K factor) prior to the IDA analysis. In this work, the K factor is defined as K = R_true_/R_measured_, where R_true_ = (^107^Ag/^109^Ag)_natural_ = 1.075. A set of Ag ionic solutions of 2.5, 5.0, 7.5 and 10.0 µg L^−1^ with natural isotope composition was used for K factor calculation. These solutions were prepared and measured daily, and the K factor was obtained as the median of the values calculated for each of the measured solutions. All the solutions employed for this purpose were prepared in 1 mM citrate media.

#### 2.3.4. Determination of Transport Efficiency

As will be discussed in Section 3.1, determination of transport efficiency is needed for IDA calculations in spICP-MS. Pace et al. examined three methods for transport efficiency determination [7], all with advantages and disadvantages, analysis of which is outside the scope of this paper. In this work, the most direct means to determine TE based on the use of an NP suspension with a known number-based concentration was deployed (method 2 in the work by Pace). This method correlates the pulse frequency of an NP dispersion obtained by spICP-MS with the known particle number concentration of the NP dispersion.

On the other hand, it is well-known that transport efficiency is affected by the composition of the matrix [33], and thus, an appropriate matrix-matching is mandatory for the determination of this parameter with any of the existing methods [8]. As a consequence, matrix-matching between the analyzed NP suspensions and the standard NP suspensions used for TE determination was always deployed when required. The TE was determined daily prior to the IDA analysis of isotopically enriched AgNP-spiked suspensions. Five replicates were taken for the TE calculation, and the median value was used for the IDA calculations.

In those situations where a matrix-matched approach would not be feasible, TE can always be calculated via an indirect method, such as the dynamic flow approach, without the need to have any NP reference material [32].

#### 2.3.5. Preparation and Analysis of AgNP Suspensions Spiked with ^109^Ag Isotopically Enriched Solutions

To calculate the dilution factor needed for the NP suspensions analyzed, probability calculations based on Poisson statistics were carried out, taking into account the exact experimental parameters finally selected for the measurement (integration time, transport efficiency and sample uptake rate) to ensure that the probability of double events occurring was below 5% (“double event” means two NPs detected in a single integration time) [12]. According to these calculations, the ideal particle number concentration of the AgNP diluted dispersions used for IDA analysis was, approximately, 4.50 × 10^7^ particles L^−1^. The AgNP stock dispersion was vigorously shaken prior to preparation of all the NP dilutions. Each diluted AgNP dispersion was spiked with a proper dilution of the ^109^Ag isotopically enriched solution described in Section 2.1 to reach a final Ag ionic concentration of 2.0 µg L^−1^. All the AgNP-spiked dilutions were daily prepared in 1 mM citrate media and were vigorously shaken prior to analysis. Each dispersion of AgNPs with enriched spike was measured five times per batch. To prevent sample and spike carryovers occurring during the measurements, which would lead to biased results, it is imperative to include a rinsing cycle after each replicate consisting of 1 min rinse with 10% nitric acid and 2 min rinse with deionized water. Three batches were measured on three consecutive days. Five independently prepared samples were measured per batch.

#### 2.3.6. Data Acquisition and Treatment

Continuous mode was selected for data acquisition (see Section 3.2.1 for more details of this acquisition mode). For all data points on each ToFMS time trace, complete mass spectra are recorded along with integrated data from each isotope. These data are stored in the instrument and can be processed after the acquisition has finished, using the standard ToFMS acquisition software, the Tofware software (version 3.2) and in-house spreadsheets and scripts. The procedure we followed is described below.

Recalibration of mass spectra was performed using 2.5 µg L^−1^ Ag ionic standards, as previously described. Moreover, baseline fitting and correction was also performed after data acquisition. The baseline in ICP-ToFMS is a complex function of overlapping peak shapes and the contribution of stray ions from the most abundant species in the plasma. As ions of all *m/z* are measured in each ToF extraction, by design, intense peaks that originated from the matrix can result in an elevation of the baseline by several orders of magnitude [28]. Under these circumstances, baseline fitting and subtraction seems imperative, especially if complex matrices are introduced in the ICP-ToFMS instrument. For baseline fitting, a smoothing function that takes the rolling minimum of a defined number of ToF data points was selected [28].

Following data acquisition in Tofware software (version 3.2), a file containing the time of analysis and only the signals for *m/z* 107 and 109 was obtained. Before applying the IDA equations to the isotope ratios of each sample, background correction was carried out for each sample. Considering that all NP dispersions are spiked with the enriched isotopic standard, a different blank solution was measured for this purpose. The average background for each Ag isotope determined from this solution was subtracted for each individual value per integration time in each NP sample.

The corrected mass-spectral signals were processed with an Excel spreadsheet using Equations (1)–(3) from Section 3.1. Considering that the isotopically enriched spike is mostly ^109^Ag, the trace at *m/z* 107 is used to identify NP signals. For this purpose, a frequency–intensity histogram is built for the ^107^Ag isotope, and a threshold is applied for separating NP signals from that of the background. The threshold follows the 5σ criteria, where σ is defined by the *m/z* = 107 background standard deviation [34]. Next, ^107^Ag/^109^Ag isotope ratios are calculated for each event that matches the threshold condition. The IDA equation (Equation (3)), described in Section 3.1, is then applied for each isotope ratio, providing the particle mass for each detected event. As an additional measure, a script for the correction of split events was written in-house in GNU Octave (Free Software Foundation, Boston, MA, USA). With this script, NP signals that fall into two consecutive time events are integrated. The corrected particle mass is then reintroduced in the IDA spreadsheet to finally provide the NP diameter, assuming spherical form and density of bulk silver (10.49 g cm^−3^).

## 3. Results and Discussion

### 3.1. Isotope Dilution Equations for spICP-MS

Considering that the analytical signal in spICP-MS shows a transient nature, an isotope dilution equation adapted to online work was used for calculations. In particular, the basic equation for postcolumn species-unspecific isotope dilution (Equation (1)), first proposed by Rottmann and Heumann [25,26] and described in detail by Rodríguez-González et al. [20], was used as a model.

(1) $$c_{S}d_{s}f_{s}=c_{sp}d_{sp}f_{sp}\frac{M_{s}}{M_{sp}}\left(\frac{R_{m}A_{sp}^{b}-A_{sp}^{a}}{A_{s}^{a}-R_{m}A_{s}^{b}}\right)$$

The original equation is designed to quantitatively determine the different species in a mixture, which are separated in a chromatographic column. After the separation, the effluent (sample) with a density *d_s_* (ng mL^−1^) and containing a concentration of the element of interest *c_s_* (ng g^−1^) is pumped at a flow rate *f_s_* (mL min^−1^) and mixed with the spike solution. The latter shows a density *d_sp_* (ng mL^−1^), contains a concentration of the element of interest *c_sp_* (ng g^−1^) and is pumped at a flow rate *f_sp_* (mL min^−1^). As for the rest of the parameters in Equation (1), they are as follows:

- *Ms* and *Msp* are the atomic weights of the element in the sample and the spike.
- $A_{s}^{i}$ and $A_{sp}^{i}$ are the isotope abundances for the isotope i in the sample and the spike.
- Rm is the isotope ratio a/b in the mixture, which varies with time as a result of the chromatographic separation.

The expression $c_{S}d_{s}f_{s}$ is expressed in ng min^−1^ and represents the mass flow of the sample eluting from the column, *MF_s_*, which also changes with time as a consequence of the varying element concentration after the chromatographic column. Quantification of the different species can be performed by simple integration of the different peaks in the mass flow chromatogram (*MF_s_* vs. time).

In the particular case of spICP-MS, each species of the analyte (the ionic form and the nanoparticulate form) provides a different signal. Each NP event can be seen as a “chromatographic” peak in the mass flow “chromatogram”, so that Equation (1) can be used with little modification. The determined *MF_s_* obtained from Equation (1) for each NP event can be converted into the mass of the detected NP (*m_NP_*) according to Equation (2) after introducing the transport efficiency *η* and the dwell time (integration time in the case of ToF mass spectrometers, *t_int_*) as additional parameters:

(2) $$m_{NP}=\eta \cdot t_{int}\cdot {c_{s}{\cdot d}_{s}\cdot f}_{s}$$

As no chromatographic column is needed for the separate detection of the NPs and the dissolved material, online addition of the spike is not strictly necessary for spICP-MS. Although online addition of the spike might have some advantages in terms of sample preparation, batch spiking of sample aliquots might favor spike–sample equilibration, thus reducing the uncertainty of the results [19]. For this reason, batch spiking of sample aliquots was used throughout this study. The final IDA equation used for calculations (Equation (3)) combines Equations (1) and (2) and considers batch spiking of an aliquot of the NP dispersion, giving a final concentration of the spike in the mixture *c_spike_* (ng g^−1^).

(3) $$m_{NP}=\eta \cdot t_{int}\cdot c_{spike}\cdot d\cdot f\cdot \frac{M_{s}}{M_{sp}}\left(\frac{R_{m}A_{sp}^{b}-A_{sp}^{a}}{A_{s}^{a}-R_{m}A_{s}^{b}}\right)$$

In this equation, f refers to the nebulizer solution uptake rate at which the spiked dispersion is introduced in the ICP-MS, and d refers to the density of the spiked dispersion.

From all parameters included in Equation (3), most are either user-defined ($t_{int},c_{spike},f)$ or known (d, $M_{s}$, $M_{sp}$, $A_{s}^{i}$ if a natural isotopic composition is assumed for the sample and $A_{sp}^{i}$ if a certified spike with known isotopic composition is used). The only two parameters that must be experimentally determined for IDA calculations are the transport efficiency η and the isotope ratio $R_{m}$ for each NP event. Measurement of these parameters with the best precision and accuracy is thus needed to achieve the best possible results. As discussed in the experimental section, the measurement of the transport efficiency has been extensively treated in the literature. Thus, the evaluation of the different existing methods was not targeted in this work, and only the particle frequency method described by Pace et al. [7] was carried out, matching the media for the reference NP dispersion with the sample matrix.

As for estimation of the isotope ratio for each NP event ($R_{m}$), the method development for optimizing accuracy and precision for the measurement of this parameter will be discussed in the following sections.

### 3.2. Accurate and Precise Measurement of Isotope Ratios for Individual Nanoparticle Events (Rm)

#### 3.2.1. Data Acquisition Mode in ICP-ToFMS for NP Detection

As explained in detail in the literature [28], the ICP-ToFMS instrument deployed in this work can be operated in two data acquisition modes, either continuous or trigger mode. In continuous acquisition mode, detection of the full mass spectra (7–275 amu) is possible at a rate of up to 1000 Hz (33 ToF extractions per mass spectrum). This detection speed is, however, limited by the data transfer from the analog-to-digital converter to the computer hard drive. For the particular configuration used in this work, this limitation implies a maximum time resolution of 3 ms in continuous acquisition mode.

In trigger-mode acquisition, data are only recorded if the signal level from a specified *m/z* channel is above a user-defined threshold. The time resolution in trigger mode is only limited by the repetition rate of the ToF mass analyzer (30 µs), although at the cost of only being able to record 32 data points for each triggered event. It is important to note that the selection of this acquisition mode for NP detection is only possible if a continuous signal for an isotope different from that used for NP detection can be used as a trigger, as otherwise, the signal for the BG needed for NP quantification is not recorded by the instrument. This can be easily achieved, though. In the work by Hendriks et al. [28], for instance, the NP dispersion was doped with an ionic solution of Cs, an element of no interest for the analysis, and the signal of ^133^Cs^+^ was used as trigger.

When aiming for NP detection, selecting one acquisition mode or the other is primarily affected by two opposing factors that must be evaluated by the analyst in each particular case. As extensively covered in the literature, the signals generated by NPs in ICP-MS typically show a duration of 300–500 µs [21,22], and hence, selection of integration times in the order of 50–100 µs (at present only offered by the trigger mode) would be preferred for improving size-detection limits (LOD_size_), as the contribution of the background (BG) to the signal is then minimized. On the other hand, the possibility of simultaneously detecting the full mass range is exclusive to ICP-ToFMS instruments in continuous mode, and it might justify the marginal loss of detection power derived from selection of a longer integration time when unknown samples are targeted.

In addition to these two factors, a third consideration turns out to be relevant for the case of IDA. In fact, the application of IDA equations shown in Section 3.1 requires the calculation of the isotope ratio of the mixture NP + spike for each individual NP event (*R_m_*) and, as will be shown in Section “Selection of the Integration Time and Correction for Split Events”, the precision and the accuracy of these ratios is affected by the integration time selected. In practice, shorter integration times lead to larger uncertainties for *R_m_* values. This uncertainty adds to the noise of the ICP-ToFMS measurement, which results in distorted (wider) NP size distributions if compared with “true” (as characterized by other techniques) values [28].

In this work, the continuous mode with 3 ms time resolution (spectral averaging time) was selected for evaluation of the IDA calibration methodology for the sizing of AgNPs of approximately 75 nm nominal diameter.

#### 3.2.2. Effect of Integration Time (*t_int_*) and Composition/Concentration of the Spike $\left(A_{sp}^{i},c_{spike}\right)$

The basic working principle of an ICP-ToF mass spectrometer is to inject an isokinetic packet of ions sampled from the plasma into a field-free flight tube, at the end of which is an ion detector. Isotopes of different mass-to-charge ratios travel at different speeds through the flight tube, such that they reach the detector at different moments and are, hence, separated. Even though detection occurs at different moments in time, each one of these packets of ions is sampled simultaneously, such that the level of correspondence achieved for different nuclides is very high, permitting most sources of noise related to the ICP ion source to be filtered out when isotope ratios are calculated. Under these conditions, the isotope ratio precision achievable with this type of instrument is mostly limited by counting statistics, which depends in turn on the signal intensity (in cps) and the integration time (*t_int_*) selected [28].

**Figure 1:**
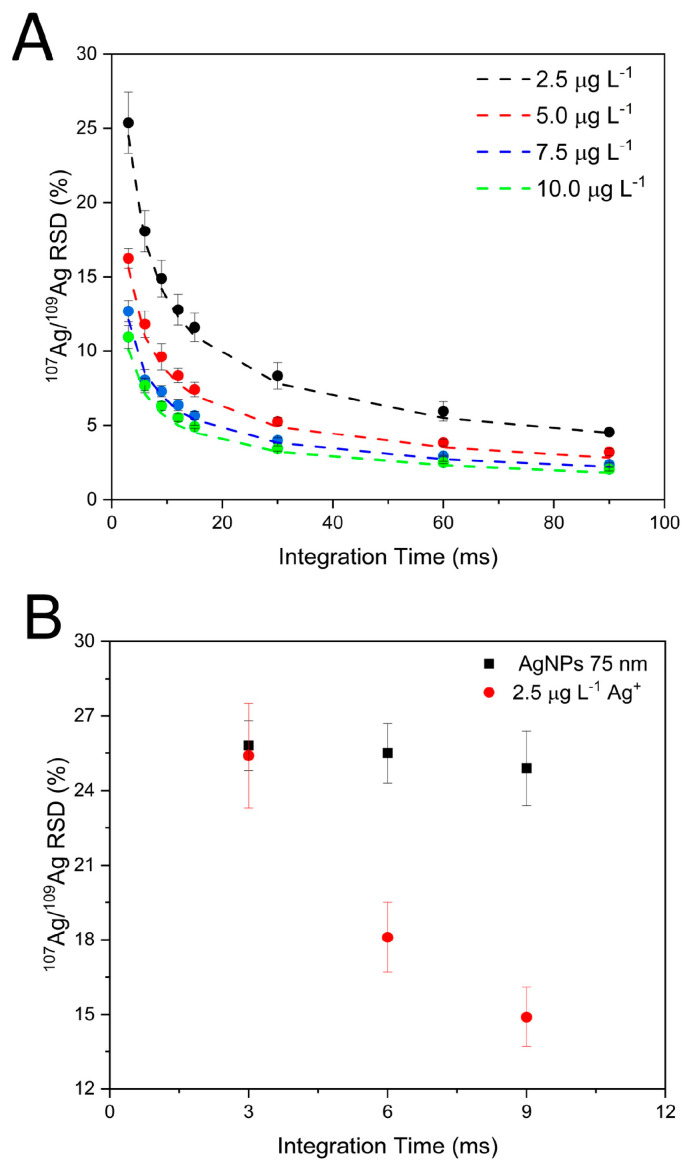
(**A**) Isotope ratio precision (% RSD) for Ag ionic solutions of different concentrations as a function of integration time. The dotted lines represent the theoretical RSD value according to counting statistics, while dots represent experimental values. (**B**) Isotope ratio precision (% RSD) for a 75 nm AgNP suspension and a 2.5 µg·L^−1^ Ag ionic solution. In both figures, error bars represent one standard deviation (*n* = 3, measured on different days).

When IDA analysis is carried out in digested samples, these parameters can be tuned to a certain extent to obtain better-precision values. Generally speaking, the longer the integration time and the higher the analyte concentration, the better the counting statistics and the isotope ratio precision. This is shown in Figure 1A, where precision values attained for ^107^Ag/^109^Ag isotope ratios for ionic silver solutions of different concentrations are displayed as a function of the integration time selected. These data were acquired at a spectral averaging time of 3 ms, while isotope signals were summed in data postprocessing to achieve the extended integration times shown in the figure. As seen in Figure 1A, isotope ratio precision improves for longer integration times and higher analyte concentration values, and in all cases, experimental data fit the expected precision reasonably well according to counting statistics [35]. A similar experiment was conducted in the work by Hendriks et al. [28] but for longer integration times, and the conclusion was that accordance to counting statistics can be expected for *t_int_* up to 100 s. From this value on, problems with mass-calibration drifts worsen the RSD.

The situation for isotope ratio determination of individual NP events is different, and analyte concentration and integration time cannot be tuned in a similar manner for optimizing precision. In fact, each NP event has a fixed duration in the 300–500 µs range [21,22] and provides a given intensity that depends on the NP size and the instrument absolute sensitivity (in counts per atom). The precision ultimately obtained is barely affected by the integration time selected at the millisecond time resolution, and increasing the NP concentration in the suspension monitored does not result in events of increased intensity (and hence better counting statistics) but in more frequent events of the same intensity. This is shown in Figure 1B, where precision values attained for ^107^Ag/^109^Ag isotope ratios for a suspension of Ag NPs of 75 nm nominal diameter are shown as a function of integration time. Data obtained for a 2.5 µg·L^−1^ Ag ionic solution of natural abundance, providing similar intensity values (in counts) for 3 ms integration time, are also displayed for comparison purposes. Every NP event provides an intensity value of approximately 40–50 counts (for the most frequent size) for each silver isotope, regardless of the integration time selected. Self-evidently, increasing the integration time does not have any effect on the isotope ratio precision for individual NPs, which remains at a constant value, corresponding well with the counting statistics prediction for the recorded signal. For the 2.5 µg·L^−1^ Ag ionic solution, the signal intensity (in counts) for each silver isotope increases with the integration time, and isotope ratio precision improves accordingly. Also, in this case, the RSD obtained corresponds well with data predicted from counting statistics.

As concluded from results shown in Figure 1, and as expected, the precision attainable for isotope ratio determination of individual nanoparticles in an ICP-ToFMS instrument is ultimately determined by the instrument absolute sensitivity (in counts per atom), and little can be done in method development to improve this value. In the case of IDA, however, *R_m_* is monitored, which includes the signal coming from the individual NP and that from the spike. The signal for the individual NP is fixed (for each size), but the signal obtained from the spike depends on the composition and concentration of the ionic spike and on the integration time. These parameters are interrelated and influence the total amount of counts recorded per event and, hence, the precision attained for *R_m_*. As explained in the following sections, we followed a straightforward approach for the proper selection of these parameters, where we first decided on the integration time (according to general considerations for spICP-MS analysis) and later adjusted the spike composition and concentration.

##### Selection of the Integration Time and Correction for Split Events

For conventional spICP-MS (calibration with external ionic solutions), and when integration (or dwell) times in the millisecond range are used, the best option seems to be selecting an integration time that is sufficiently low to minimize the background contribution to the NP signal but long enough to minimize the probability of splitting the signal of a single NP in various events [12]. Additionally, the particle number density of the NP dispersions measured is also critical in this methodology, as the probability of measuring two or more NPs in a single integration time needs to be minimized. In this regard, there tends to be an agreement in the spICP-MS community to use dwell (integration) times of 3–5 ms, as this range is the most suitable [1,36].

According to these principles, 3 ms (the minimum possible for continuous acquisition mode) was selected as the integration time in this particular case. As discussed in more detail in Section “Selection of the Isotopic Composition and Concentration of the Spike”, this value together with the selection of a ^109^Ag-enriched spike close to 100% purity ${(A}_{sp}^{109}\sim 1)$ enabled us to improve the LOD_size_ for analysis. The particle number density for the NP dispersions measured was adjusted by dilution of the original suspensions to detect between 10 and 15 particle events per second in order to minimize the occurrence of double-particle events (probability below 5%) with this integration time (see the experimental section for details).

On the other hand, and as analyzed in detail in the paper by Liu et al. [37], for integration times in the millisecond range, a significant amount of NP signals is still split into different events (split events) when such short times (below 5 ms) are selected, and implementation of a proper method for correction would be advisable. The theoretical probability of split events was calculated prior to data treatment. Equation (4) shows the probability of split events according to Poisson statistics:

(4) $$P_{split}=\frac{t_{pulse\;}{\lambda e}^{-\lambda }\;}{t_{dwell}(1-e^{-\lambda })\;}$$

where $t_{pulse\;}$ is the NP pulse duration in the plasma (s), $t_{dwell\;}$ is the dwell or, in this case, integration time (s) and *λ* is the nanoparticle detection frequency (NPs s^−1^) multiplied by the integration time (s). Considering the daily values for particle number concentration, sample flux and TE, values for $t_{dwell\;}$ (3 ms) and an average $t_{pulse\;}$ of 400 µs, the probability of split events was found to be significant, approximately 13.0%.

After carrying out all the analyses, it was indeed experimentally confirmed that, with this integration time, a significant number of NP signals were split into different events. For the identification and proper correction of these events, these signals must be integrated as one coming from a single NP, in a similar manner as performed when instruments with integration (dwell) times in the µs range are deployed. For this purpose, a script was written in GNU Octave that performs the following actions:

1. Split events identification: split events are identified when two adjacent intense signals over a user-defined threshold are detected for the *m/z* = 107 trace (where contribution of the spike to the signal is low, as composition of the spike was selected to be $A_{sp}^{109}\sim 1$). This threshold was set as the average background for the *m/z* = 107 trace, as for the acquisition conditions used in this work, the probability for two adjacent signals coming from two different NPs instead of a split one is negligible [37].
2. NP mass integration: the masses obtained for these adjacent signals after application of the IDA equation (Equation (3)) are integrated by the script.
3. NP diameter calculation: the diameter of the NP is calculated with the integrated value obtained in step (2) (see Section 2.3. for more details).

**Figure 2:**
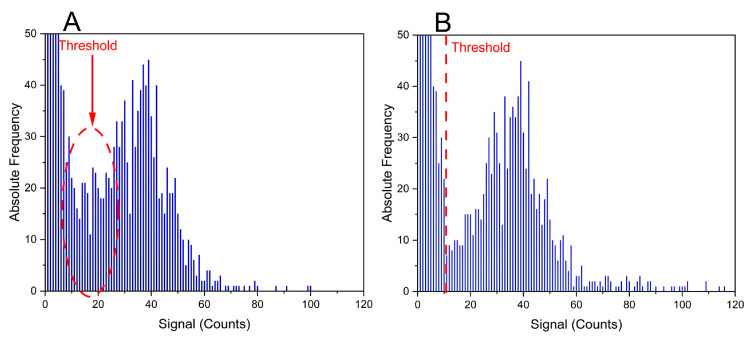
Signal distributions for ^107^Ag isotope obtained for a 75 nm nominal diameter AgNP dispersion: (**A**) without split-event correction and (**B**) with split-event correction, using the GNU octave script described in Section “Selection of the Integration Time and Correction for Split Events”.

With the described script, an average percentage of split events of 10.0 ± 0.6% was detected in the different experiments conducted in this work (*n* = 5), which corresponds well with the expected value based on Poisson probability calculations presented above. Implementing this correction script is important for correct determination of the NP size distribution, as shown in Figure 2. As seen from this figure, the occurrence of abundant split events artificially increases the number of small NPs (if compared with the reference distribution from NIST), which (i) decreases the average size of the NPs and (ii) hampers the setting of a suitable threshold for defining an NP. After correction for split events, this region is much better defined, which results in better LOD_size_.

The particle number recovery without correction was, on average, 106 ± 7%, while after correction with the GNU octave script it was 96 ± 6%. It is important to note that this correction is only effective for events providing a signal above the 5σ threshold applied for particle identification. This could be a problem for NP distributions close to the detection limit, as some small NPs might pass undetected if split in two. In such situations, the selection of an integration time in the microsecond range would be preferred for reaching the best detection capabilities. On the other hand, the selection of longer integration times would reduce the occurrence of split events, but higher dilution factors would be needed to minimize the probability of occurrence of double-particle events, the measuring time would have to be increased to compensate for such a dilution and the LOD_size_ would increase due to a larger contribution of the background to the *m/z* = 107 signal.

##### Selection of the Isotopic Composition and Concentration of the Spike

The spike isotopic composition was selected to be $A_{sp}^{109}\sim 1$ in order to minimize its contribution to the *m/z* = 107, which is used for NP identification. This permits us to improve the LOD_size_. For the same reason, the concentration of the spike would need to be kept sufficiently low. However, and considering the inherent imprecision of bond-to-isotope ratios determined for individual NPs as shown in Figure 1, it was confirmed that selecting too low a spike concentration has detrimental effects for quantitative results.

**Figure 3:**
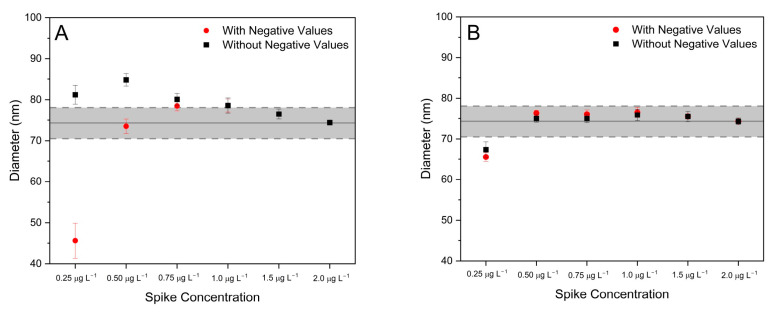
Average (**A**) and median (**B**) diameter values obtained for analysis of a 75 nm nominal diameter AgNP suspension as a function of the ^109^Ag spike solution concentration deployed for 2 different types of data treatment: with and without the consideration of negative values. Error bars represent 95% confidence intervals (*n* = 15; 5 replicates per day, 3 days of measurement). The hatched area in grey represents the reference average diameter and standard deviation obtained by TEM analysis (74.3 ± 3.8 nm), according to the certificate of analysis provided by NIST.

In fact, too-low spike concentrations result, first, in some experimental *R_m_* values above the expected natural ratio (107/109 = 1.075), which in turn result in negative mass values for the detected NPs once Equation (3) is applied. This occurrence of negative mass values was also observed by Sötebier et al. in their work [24], which they solved by just removing the negative values from the NP distributions, claiming that this measure does not affect the final results. To test the validity of this statement, IDA analysis of a AgNP dispersion doped with different amounts of the selected isotopic spike with composition $A_{sp}^{109}\sim 1$ was carried out. Results are presented in Figure 3 and Table 2. As seen from Table 2, the higher the spike concentration, the lower the percentage of negative values obtained. For concentration values above 0.75 µg L^−1^, which provide *R_m_* values of about 0.6 for the most frequent size of NP, the percentage of negative values is already negligible, which is consistent with the results shown in Figure 1. In fact, if the precision values attainable for isotope ratio determination of individual NPs are about 25–30%, then the probability for the experimental isotope ratios to exceed the natural value of 1.075 for an expected ratio of 0.6 would be rather small.

For low spike concentrations (e.g., 0.25 µg·L^−1^), on the other hand, the effect of the negative values on the sizing of the NPs is evident from Figure 3. As could be expected, if negative values are included in the distribution, an average diameter biased low is obtained. On the other hand, if negative values are just removed from the distribution as recommended by Sötebier et al., results improve, but a positive bias on the average diameter determined is observed instead. For spike concentrations above 0.75 µg·L^−1^ (providing a negligible number of negative values), the effect is much less pronounced, and no significant differences from certificate values are observed. It is interesting to note that the median values are much less affected by the presence of negative results, as seen from Figure 3B. This is not surprising considering the median is known to be a much more robust estimator in the presence of outliers.

**Figure 4:**
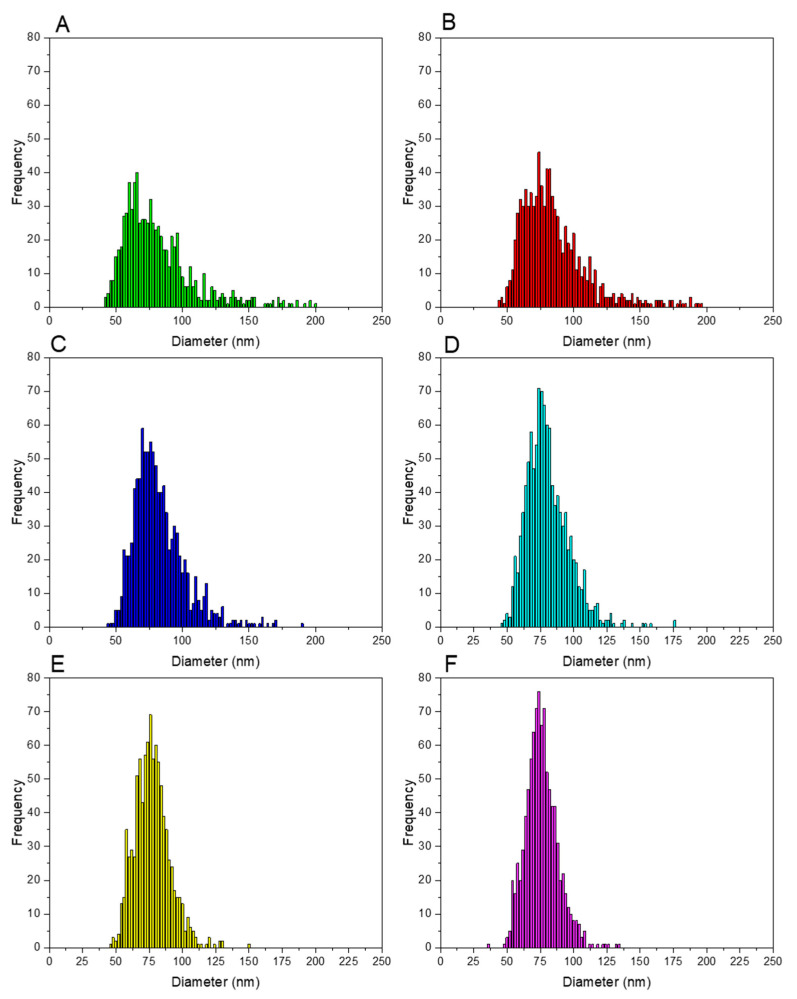
Particle size distributions obtained for the analysis of a 75 nm nominal diameter AgNP dispersion spiked with different concentrations of the ^109^Ag isotopically enriched solution: (**A**) 0.25 µg·L^−1^ spike concentration; (**B**) 0.50 µg·L^−1^ spike concentration; (**C**) 0.75 µg·L^−1^ spike concentration; (**D**) 1.00 µg·L^−1^ spike concentration; (**E**) 1.5 µg·L^−1^ spike concentration and (**F**) 2.0 µg·L^−1^ spike concentration. For distributions (**A**–**C**), negative values have been removed for the sake of simplicity.

To understand the change from negative to positive bias in the results observed, a closer look at the NP distributions obtained is needed. In fact, an additional effect is observed if spike concentration is not appropriately selected. Similar to what was indicated in Section 3.2.1 for the integration time, the selection of a lower spike concentration results in poorer counting statistics and larger uncertainties for *R_m_* values. This uncertainty adds to the noise of the ICP-ToFMS measurement, which results in distorted (wider) NP size distributions if compared with “true” (as characterized by other techniques) values [28]. This can be observed in Figure 4, where the size distributions obtained for IDA analysis of a 75 nm AgNP dispersion with different spike concentrations is presented. Negative values, if present, were removed from the histograms.

**Figure 5:**
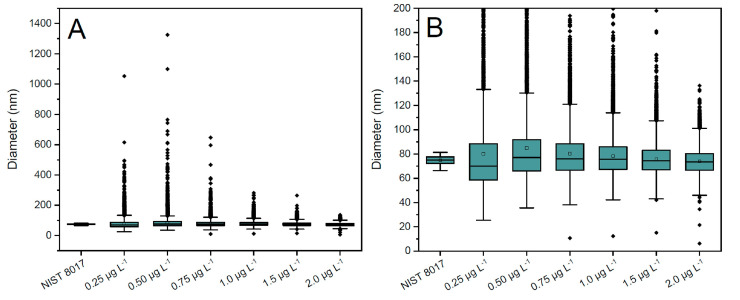
Boxplots for the analysis of a 75 nm nominal diameter AgNP dispersion (without negative values) spiked with different concentrations of the ^109^Ag isotopically enriched solution (2 days of analysis). The thick horizontal line across each box marks the median of the corresponding particle size distribution, the square dot inside the box represents the mean and the bottom and top of the box indicate the 25th and 75th percentiles, respectively. The bottom and top whiskers indicate the range of data within 1.5 times the interquartile range. Individual diamonds represent values outside 1.5 times the interquartile range (outliers). The boxplot labelled NIST 8017 represents data gathered in the NIST report of analysis, for which individual data outside 1.5 times the interquartile range are not available. (**A**) Complete representations, including outliers. (**B**) Zoom-in of A in order to compare all the median, mean, 25th and 75th percentiles individual values.

As seen in Figure 4, the lower the spike concentration, the wider the NP distribution obtained, and the effect is more pronounced to the right wing of the distribution. This is more evident in Figure 5, which includes boxplots for all the NP distributions obtained with IDA analysis and different spike concentrations plus the one obtained with external calibration and that provided by the NIST certificate of analysis, included for comparison purposes. As seen from this figure, narrower distributions are obtained as the spike concentration increases. For a spike of 2.0 µg·L^−1^, it can be observed that there is no practical difference between the average and the median, thus indicating the negligible influence of outliers. This fact is also supported by the varying mass concentration recoveries obtained for each distribution, which are gathered in Table 3. In that table, the results are given as the median value of the measurements for 3 days. On the third day of measurements, the results of particle mass concentration obtained were considerably higher than those obtained on the other two days of measurements. Thus, in this case, the median displays a better observation of the particle mass recovery than the average value. The average value of the first two days is also provided for comparison.

As seen in Table 3, the low spike concentrations result in significant positive bias for the mass concentration recovery, which is consistent with the enlargement of the distribution towards the larger diameter values seen in Figure 4 and Figure 5. For 2.0 µg·L^−1^, the mass concentration recovery is very close to 100% (98% for both the average and the median), which serves as an indicator that such a spike concentration the most suitable one.

As for the spICP-MS NP distribution, the comparison with the data provided by NIST and gathered in Figure 5 cannot be complete as no individual values are provided in the certificate of analysis. However, it seems that the NP distributions obtained by spICP-ToFMS (from either external calibration or IDA) are wider than those reported by NIST, especially towards larger diameters. However, it has to be mentioned in this regard that, according to the literature [38], relatively large differences in NP distribution data are common for different analytical techniques or even different operators, such that data provided on mass concentration recovery can be considered as a good indicator of the quality of the results.

Finally, the influence of the spike concentration on the LOD_size_ was evaluated. As indicated above, increasing spike concentrations could result in higher background for *m/z* = 107, which is used for NP identification. This would degrade LOD_size_. In this work, LOD_size_ was calculated for the different spike concentrations used in this study following the equation proposed by Laborda et al. [1]. A summary of the LOD_size_ obtained is included in Table 4. As seen from this table, there are no significant differences in LOD_size_ for the spike concentrations deployed in this work, such that selection of the spike is better achieved in terms of the shape for the NP distribution obtained, as explained above. Accordingly, a spike concentration of 2.0 µg·L^−1^ was used for further analysis in this study.

#### 3.2.3. Mass-Discrimination Correction

It is well-known that heavier isotopes are better transmitted in ICP-MS instruments. This mass-discrimination effect is known to be produced in the interface (nozzle effect) or in the ion optics (space-charge effect) [20].

Among the different methods existing for mass-bias correction, the sample-standard bracketing method was considered to be the most appropriate in this case. It has been documented that there is a mass peak drift in ICP-ToFMS when integration times are long [28], so it is important to mass-calibrate the instrument often. In the sample-standard bracketing method, a mass-bias correction factor, K, is calculated as K = *R_true_*/*R_measured_* for a standard with well-known or certified isotopic composition, and this factor is later applied to the sample measured ratios to obtain the true values. For elements that do not show significant natural variations in their isotopic abundances, such as Ag, any standard (with natural isotopic composition) can be used for correction. For this particular case, moreover, a dispersion of AgNPs of natural isotopic composition can be also used for correction. Calculation of the K factor was carried out in this work with these two options: a 75 nm AgNP dispersion on the one hand, and a set of ionic solutions with concentrations of 2.5; 5.0; 7.5 and 10.0 µg L^−1^ on the other. Results are compared in Table 5. As seen from this table, there are no significant differences in the median K values obtained with one option or the other. For the ionic standards, the uncertainty is lower than for measurements of the AgNP dispersion; this was due to the possibility of measuring with longer integration times (100 ms). As a result, the calculation of the K factor was carried out with the ionic standards in all further IDA experiments.

### 3.3. Application of the IDA Developed Method for Sizing AgNPs Using ICP-ToFMS

**Figure 6:**
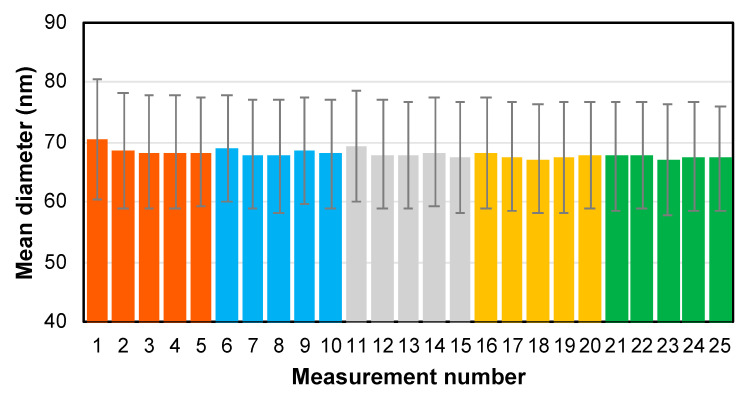
Example of within-batch precision of the results obtained for the sizing of the NIST RM 8017 (AgNP) by means of IDA spICP-ToFMS (results presented correspond to Batch 2 from Table 6). Error bars show one standard deviation for the nanoparticle size distribution for each replicate measurement. Different colors indicate the five different samples analyzed.

After method optimization, the final protocol was applied for sizing the NIST RM 8017 (AgNP) material. Analysis was performed in three different batches that were measured on three consecutive days. Each batch consisted of the analysis of five independent sample dispersions, each one measured in quintuplicate. It is important to note that these measurements were performed several months after the optimization was carried out, due to instrument unavailability. The protocol previously optimized was applied as such, without further reoptimization, so that information on the method robustness could be evaluated. Results obtained are gathered in Table 6. Also, an example of the precision obtained within each batch is shown in Figure 6.

As has already been commented on and as shown in Table 6, slightly different values are typically obtained for the different analytical techniques used for sizing nanoparticles, although the results obtained by the IDA ICP-ToFMS approach are in good agreement with most of the results presented in the certificate of analysis for the NIST 8017 AgNPs reference material. The uncertainty obtained for the IDA ICP-ToFMS results is of similar magnitude or better than for the techniques based on NP deposition on substrate (AFM and TEM). As for the techniques based on measurements of suspensions, USAXS results have a lower associated measurement uncertainty, but this technique requires concentrated suspensions, while DLS results seem to be biased high in comparison with the other techniques. One of the advantages of spICP-MS is the possibility to perform the analyses in highly diluted suspensions, which might be relevant in environmental studies, among other applications that might involve analyses of complex samples. The IDA approach presented in this work offers the added advantage of the reduction in the effect of potential matrix effects on the accuracy of the data.

## 4. Conclusions

In this work, a method for the accurate mass determination of AgNPs by means of isotope dilution spICP-ToFMS has been optimized and used for the sizing of NIST RM 8017 AgNPs. The use of a ToF mass analyzer permitted the measurement of isotope ratios in a particle-to-particle approach. It was shown that the precision of the measurement of isotope ratios of individual NPs is limited by the magnitude of the signals obtained for each NP in the mass analyzer, and hence by the instrument absolute sensitivity (counting statistics). However, the uncertainty obtained for the sizing of NPs by the IDA approach can be improved by careful method optimization, where the most important parameters were shown to be the selection of the spike isotopic composition and concentration. Other parameters such as integration time or ToF acquisition mode typically affect LOD_size_ attainable and/or occurrence of double events in the same manner as they affect any spICP-MS method deploying other calibration strategies. The method optimized in this work (with an integration time of 3 ms) favors precision over LOD_size_ attainable, in which case other optimization strategies should be explored. Although only AgNPs were targeted in this study, the IDA method presented, with the corresponding adaptations, could be applied to NPs of any other composition that include an element with different naturally available isotopes.

Finally, the IDA spICP-ToFMS approach presented for the first time in this paper offers the possibility to size NPs in highly diluted suspensions, which might be relevant in, e.g., environmental studies or other applications involving analyses of complex samples. In particular, the use of IDA has the potential advantage of reducing the impact of matrix-induced effects on the accuracy of the data.

## Figures and Tables

**Table 1:** ICP-ToFMS working parameters.

Component/Parameter	Type/Value/Mode
Plasma gas flow rate	14.0 L min^−1^
Auxiliary gas flow rate	0.80 L min^−1^
Nebulizer gas flow rate	1.05 L min^−1^
RF power	1550 W
Integration time	3 ms
Data acquisition mode	Continuous mode
Collision/reaction cell gas	Not used
Data acquisition isotopes	^107^Ag, ^109^Ag
Detected mass range	^23^Na-^238^U
Total acquisition time per sample	1 min

**Table 2:** Percentage of negative values and average *R_m_* values (^107^Ag/^109^Ag) obtained (standard deviation of 3 days of analysis) for analysis of a 75 nm AgNP suspension by IDA as a function of the spike concentration deployed.

Spike Concentration (µg L^−1^)	Negative Values (%)	Average *R_m_* ± SD
0.25	14.2	0.8633 ± 0.051
0.50	3.4	0.7167 ± 0.020
0.75	0.4	0.5969 ± 0.012
1.00	0.1	0.5217 ± 0.0069
1.50	0.0	0.4063 ± 0.0065
2.00	0.0	0.3217 ± 0.0019

**Table 3:** Particle mass recovery for each spike concentration (median values for the three days of analysis and average ± standard deviation of the first two days of analysis).

Spike Concentration (µg L^−1^)	Particle Mass Recovery (Median) (%)	Particle Mass Recovery (Average ± SD) (%)
0.25	338	270 ± 96
0.50	293	373 ± 159
0.75	168	167 ± 36
1.00	142	138 ± 6.0
1.50	119	112 ± 10
2.00	98	98.0 ± 0.1

**Table 4:** LOD_size_ by each spike condition (standard deviation of 2 days of analysis).

Spike Concentration (µg L^−1^)	LOD_size_ ± SD (nm)
0.25	33.8 ± 1.7
0.50	33.1 ± 0.80
0.75	32.6 ± 0.57
1.00	32.7 ± 0.50
1.50	32.4 ± 0.82
2.00	32.3 ± 0.74

**Table 5:** Median K factor obtained after measurement of a 75 nm nominal diameter AgNP dispersion and for a set of Ag ionic solutions of 2.5, 5, 7.5 and 10 µg·L^−1^. Confidence intervals represent one standard deviation of 3 different days.

Sample	K Factor ± SD
NPs	1.0562 ± 0.0126
Ionic solutions	1.0554 ± 0.0033

**Table 6:** Average and median AgNP size determined via IDA ICP-ToFMS for three batches, compared to the reference size values from the NIST RM 8017 certificate.

	Batch 1	Batch 2	Batch 3	Average of Batches (nm) ± Expanded Uncertainty (k = 2)	Reference Values (nm) ± Expanded Uncertainty (k = 2) ^1^			
					AFM	TEM	USAXS	DLS
Average AgNP size (nm)	69.6	69.3	70.3	69.8 ± 2.7	70.1 ± 6.0	74.6 ± 3.8	67.9 ± 0.8	105.6 ± 4.6
RSD (%)	0.31	0.48	0.84					
Median AgNP size (nm)	69.8	69.4	70.7	69.8 ± 2.7				
RSD (%)	0.28	0.67	1.40					

^1^ NIST RM 8017 certificate.

## Data Availability

Data will be available through Zenodo.
